# Dimethyl Sulfoxide-Free Cryopreservation of Human Umbilical Cord Mesenchymal Stem Cells Based on Zwitterionic Betaine and Electroporation

**DOI:** 10.3390/ijms22147445

**Published:** 2021-07-12

**Authors:** Lei Gao, Qianqian Zhou, Yulong Zhang, Sujing Sun, Liping Lv, Ping Ma, Jing Yang, Min Liu, Lei Zhang, Xiaohui Wang, Linsheng Zhan

**Affiliations:** 1 Department of Emerging Transfusion Technology, Institute of Health Service and Transfusion Medicine, Academy of Military Medical Sciences, Beijing 100850, China; GL8023jc@163.com (L.G.); zhouqianvv521@126.com (Q.Z.); Zhangyulongklg@163.com (Y.Z.); ammsreb@163.com (S.S.); Lvlp2007@sina.com (L.L.); Maping1111@Hotmail.com (P.M.); 2 Department of Biochemical Engineering, School of Chemical Engineering and Technology, Tianjin University, Tianjin 300350, China; jing_yang@tju.edu.cn (J.Y.); minliu@tju.edu (M.L.); 3 Frontier Science Center for Synthetic Biology and Key Laboratory of Systems Bioengineering (MOE), School of Chemical Engineering and Technology, Tianjin University, Tianjin 300350, China; 4 Qingdao Institute for Marine Technology of Tianjin University, Tianjin University, Qingdao 266235, China; 5 Key Laboratory of Advanced Energy Materials Chemistry, Ministry of Education, College of Chemistry, Nakai University, Tianjin 300071, China

**Keywords:** betaine, bioluminescence imaging, cryoprotectant, molecule delivery, reactive oxygen species

## Abstract

The effective cryopreservation of mesenchymal stem cells (MSCs) is indispensable to the operation of basic research and clinical transplantation. The prevalent protocols for MSC cryopreservation utilize dimethyl sulfoxide (DMSO), which is easily permeable and able to protect MSCs from cryo-injuries, as a primary cryoprotectant (CPA). However, its intrinsic toxicity and adverse effects on cell function remain the bottleneck of MSC cryopreservation. In this work, we cryopreserved human umbilical cord mesenchymal stem cells (UCMSCs) using zwitterionic betaine combined with electroporation without any addition of DMSO. Betaine was characterized by excellent compatibility and cryoprotective properties to depress the freezing point of pure water and balance the cellular osmotic stress. Electroporation was introduced to achieve intracellular delivery of betaine, intending to further provide comprehensive cryoprotection on UCMSCs. Compared with DMSO cryopreservation, UCMSCs recovered from the protocol we developed maintained the normal viability and functions and reduced the level of reactive oxygen species (ROS) that are harmful to cell metabolism. Moreover, the in vivo distribution of thawed UCMSCs was consistent with that of fresh cells monitored by a bioluminescence imaging (BLI) system. This work opens a new window of opportunity for DMSO-free MSC cryopreservation using zwitterionic compounds like betaine combined with electroporation.

## 1. Introduction

Mesenchymal stem cells (MSCs) have emerged as the ideal candidate for treatments of tissue damage, graft versus host diseases, osteoarthritis, and gynecological diseases due to their favorable properties such as self-renewal, multi-lineage differentiation, and immunoregulation [1,2]. The success of these clinical applications requires a large number of viable MSCs [3]. Fresh MSCs isolated from bone marrow, adipose tissue, umbilical cords, and other tissues are limited, however, and must be pooled in vitro. Continuous expansion of MSCs in vitro consumes substantial resources and may result in heterogeneity or tumorigenicity. Cryopreservation is currently the only reliable technology for long-term storage of cells [4]. At cryogenic temperatures (−150~−196 °C), all cellular metabolism can be ‘suspended’. After being warmed in a water bath (37 °C), the cryopreserved cells recover with maintained viability and functions. MSC cryopreservation is indispensable to ensure that the manufacturing, distribution, and unpredictable demands of MSCs can be addressed [5,6]. The current protocols for MSC cryopreservation require dimethyl sulfoxide (DMSO), which is easily permeable and able to protect MSCs from cryo-injuries [7]. However, DMSO is systemically toxic in humans and must be rapidly removed after cell rewarming [8]. It has been reported that approximately 1 in 70 transplants experience DMSO-related complications through investigating the data of 444 European Cooperative Group for Bone Marrow Transplantation (EBMT) centers and most cases were cardiovascular and respiratory in nature. The trace amounts of DMSO even after wash step remained high risk to trigger adverse reactions in patients [9]. Moreover, DMSO has been suggested to be associated with abnormal gene expression and differentiation of MSCs [10]. Clearly, the development of DMSO-free cryopreservation of MSC is desirable.

To address the challenges faced in MSC cryopreservation, a large number of biocompatible materials have been investigated to replace DMSO. Trehalose [11], plant proteins [12], and mimics of anti-freezing proteins [13,14] have been combined with a reduced concentration of DMSO for enhanced MSC cryopreservation. In particular, carboxylated poly-L-lysine (COOH-PLL) demonstrated high potency for cryopreservation of various MSCs, such as mouse MSCs (>90%) [15] and human MSCs (>90%) [16]. COOH-PLL was able to inhibit ice recrystallization and protect cell membranes in a manner similar to that of the natural antifreeze protein [15]. Advanced biotechnologies such as microfluidic-based hydrogel microencapsulation, droplet-based cell printing, and nanowarming have also been applied to improve MSC cryopreservation [17]. MSC vitrification with low concentration of cryoprotectant (CPA) was achieved by introducing microfluidic-based hydrogel microencapsulation or droplet-based cell printing technology [18,19,20]. Nanomaterials mediated by nanowarming technology overcame the drawbacks of the conventional water bath (37 °C) warming method and allowed for an ultra-rapid and uniform warming process [21,22]. However, these advances in MSC cryopreservation ignored the influence of the newly established methods on the in vivo characteristics of MSCs. The distribution, migration, and homing properties of MSCs are the foundation of MSCs-based implantation therapy, which is associated with the cytoskeleton, adhesion molecules, and recognition of chemokines and receptors [23,24]. It has been reported that cryopreservation impaired the cytoskeleton and altered the in vivo distribution of human MSCs [25,26].

Betaine is a kind of natural zwitterionic molecule and shows a wide distribution within phylogenetically distant organisms from microorganisms to animals. It is characterized by stability, nontoxicity, and superior hydrophilicity [27,28]. In aqueous solution, betaine can competitively bind water molecules and impede the hydrogen bond network (HBN) formation, suppressing ice formation [29]. As a natural osmoprotectant, betaine can be absorbed or released by cells to adapt the external osmotic pressure [30]. Besides, betaine plays a significant role as a chemical chaperone to modify the structure and stability of macromolecules such as proteins and DNA [31]. This potentials supported its selection as a novel cell cryopreservation CPA. However, for specific cell types like chondrocytes, stem cells, and dendritic cells, betaine alone cannot work well because of insufficient intracellular cryoprotections [32].

Electroporation has been studied for more than 40 years and has been used for the intracellular delivery of non-permeable or low permeable exogenous molecules such as DNA vaccines, plasmids, proteins, saccharides, and drugs [33]. It has gained wide acceptance due to its excellent advantages including nonspecific, controllable, reproducible, and particularly non-toxic effects on cells [34]. Under the external electric stimulus, hydrophobic pores will be formed on the cell membrane that allow molecules of a smaller size than the pores to pass into cell, which can be reversible when the electroporation conditions are controlled within a reasonable range [35].

In this work, we investigated the use of betaine combined with electroporation to achieve DMSO-free cryopreservation of human umbilical cord mesenchymal stem cells (UCMSCs). In addition to the post thawed viability, the in vitro characters including proliferation ability, differential potential, expression of MSC markers, and reactive oxygen species (ROS) level and in vivo distribution of UCMSCs were evaluated to verify the effectiveness of the novel cryopreservation method.

## 2. Results

### 2.1. Cryoprotective Properties and Cytotoxicity of Betaine

**Figure 1:**
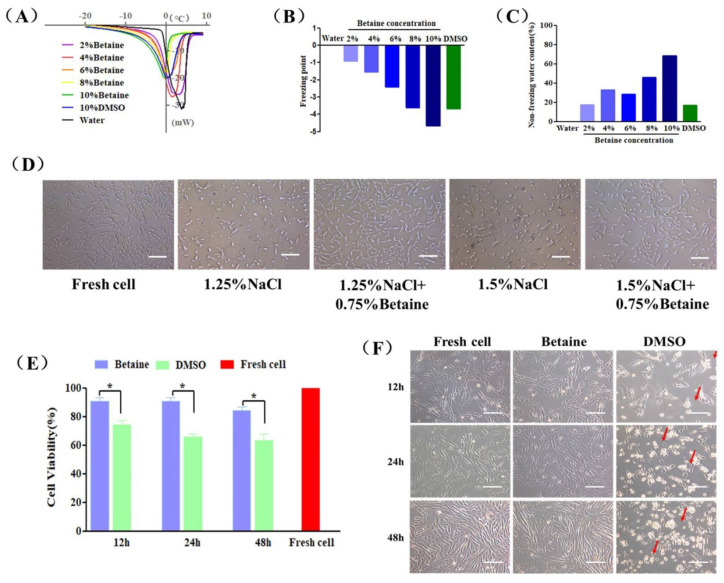
Cryoprotective properties and cytotoxicity of betaine. (**A**) DSC melting thermograms, (**B**) freezing point and (**C**) non-freezing water content of pure water (Water), betaine solutions (2–10%, *w*/*v*) and DMSO solution (10%, *v*/*v*). (**D**) The morphology of UCMSCs exposed in culture medium containing 1.25% NaCl (*w*/*v*), 1.25% NaCl (*w*/*v*) + 0.75% betaine (*w*/*v*), 1.5% NaCl (*w*/*v*), 1.5% NaCl (*w*/*v*) + 0.75% betaine (*w*/*v*) for 24 h, and culture medium (Fresh cell) as a control. (**E**) Cell survival rate and (**F**) microscopic images of UCMSCs incubated with culture medium (Fresh cell), 2% betaine (Betaine) or 2% DMSO (DMSO) for 12, 24, and 48 h. The red arrows mark deformed or floating UCMSCs. Scale bar = 200 μm. Value = mean ± standard deviation, n = 3. * *p* < 0.05.

The influence of betaine on water behaviors during the freezing and warming process was investigated by using a differential scanning calorimeter (DSC) [36,37,38,39]. Figure 1A shows that only one endothermic peak appeared in the thermograms of the examined samples due to the melting of the solute-water system. The peak value represented the freezing point of the solution. The freezing point of 10% betaine and 10% DMSO was −4.68 °C and −3.68 °C lower than the freezing point of pure water. This decreased effect of betaine was inversely proportional to the concentrations. The same trend could be observed in the freezing point of sample solutions showed in Figure 1B, as 10% betaine solution possessed a lower freezing point than 10% DMSO. On the contrary, the non-freezing water content of betaine solution increased as the concentration increased in Figure 1C.

To study the osmotic regulation ability of betaine, UCMSCs were exposed to culture medium containing 1.25% or 1.5% NaCl (hypertonic condition) at physiological temperature for 24 h. As shown in Figure 1D, after incubation, most cells severely shrank due to the loss of intracellular water, and even deformed and lost their attachment ability. Interestingly, the addition of 0.75% betaine in NaCl solution maintained normal cell morphology and adhesion.

**Figure 2:**
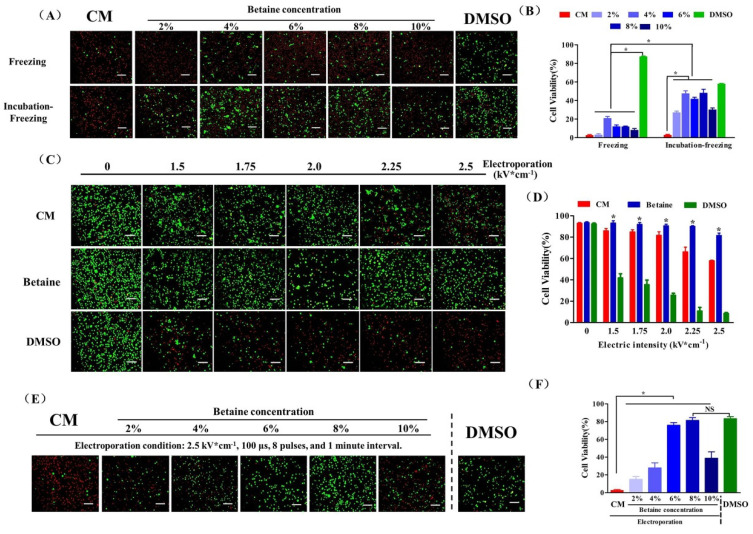
UCMSC cryopreservation. (**A**) Fluorescence images and (**B**) cell survival rate of UCMSCs cryopreserved using culture medium (CM), 2–10% betaine solution (betaine), or 10% DMSO solution (DMSO). (**C**) Fluorescence images and (**D**) cell survival rate of UCMSCs electroporated with culture medium (CM), 8% betaine solution (*w*/*v*) (Betaine), or 8% DMSO (*v*/*v*) (DMSO) as buffer in the range of electric intensity from 0 to 2.5 kV × cm^−1^. *: Betaine group compared with culture medium group and DMSO group. (**E**) Fluorescence images and (**F**) cell survival rate of UCMSCs recovered from B–E cryopreservation (CPA solution: culture medium, CM; 2–10% betaine solution, Betaine; Electroporation condition: 2.25 kV × cm^−1^, 100 μs, 8 plus and 1 min interval) or DMSO-based cryopreservation (DMSO). Green, live cells; red, dead cells. Scale bar = 200 μm. Value = mean ± standard deviation, n = 3. * *p* < 0.05, NS: no significance.

The cytotoxicity of betaine and DMSO on UCMSCs was tested by a co-incubation experiment. Figure 1E,F showed that UCMSCs incubating with 2% betaine at 37 °C for 12, 24, and 48 h maintained viabilities of 90.87 ± 4.65%, 90.62 ± 4.43%, and 84.39 ± 4.60%, respectively, and normal morphology. On the contrary, DMSO was toxic and resulted in a drastic decreases in viability of 74.16 ± 5.12%, 66.04 ± 3.33%, and 63.37 ± 7.40%, respectively. Moreover, the detachment and severe deformation (red arrow) in DMSO-incubated UCMSCs can be observed in Figure 2F.

### 2.2. UCMSC Cryopreservation

At first, we chose betaine as the sole CPA to cryopreserve UCMSCs combined with a conventional freezing protocol. As shown in Figure 2A,B, the post thaw viability of UCMSCs recovered from cryopreservation with increased betaine concentration showed an apparent elevation compared to UCMSCs cryopreserved without betaine. Before cell cryopreservation, incubating the UCMSC-betaine suspension in a CO_2_ incubator for 4 h apparently increased the post-thaw viability to about 50%, which suggested a considerable cryoprotection of betaine for UCMSCs. However, the results remained a long way away from the efficiency of DMSO cryopreservation. The electroporation procedure was introduced to further improve the efficiency of betaine for UCMSCs cryopreservation. A pulsed electric field of sufficient amplitude can cause the formation of hydrophilic pores on membranes and improved permeability. However, if the electric field is too intense, the cells can not recover their membrane integrity and functions [33]. Therefore, we first evaluated the influence of electric intensity on the viability of UCMSCs. As shown in Figure 2C,D, the cell viability is negatively correlated with electric intensity. Moreover, the composite of electroporation buffer also influenced the post-electroporation cell viability. Culture medium could not maintain post-electroporation cell viability well as the electric intensity exceeded 2 kV × cm^−1^. The addition of 8% betaine ensured a 90.06 ± 0.76% cell survival rate even with an electric intensity of up to 2.25 kV × cm^−1^. In contrast, the addition of 8% DMSO caused a great loss of cell viability.

On the premise of ensuring the cell survival rate and maximizing the intracellular content of betaine, we chose the electric intensity of 2.25 kV × cm^−1^ to operate the following experiments. As shown in Figure 2E,F, UCMSCs electroporated without betaine almost all died after cryopreservation. The increased post thaw viability of UCMSCs was observed when the betaine concentration increased from 2% to 8%. Moreover, 8% betaine solution as CPA combined with electroporation (condition: 2.5 kV × cm^−1^, 100 μs, 8 pulses, and 1 min interval) enabled a cell survival rate of 83.45 ± 2.12%, which was similar to that of DMSO-based cryopreservation. Notably, increasing betaine concentration to 10% reduced the post thaw viability, suggesting there would be a maximum concentration of betaine inside the cell, and much more extracellular betaine would cause serious osmotic injury for cells.

**Figure 3:**
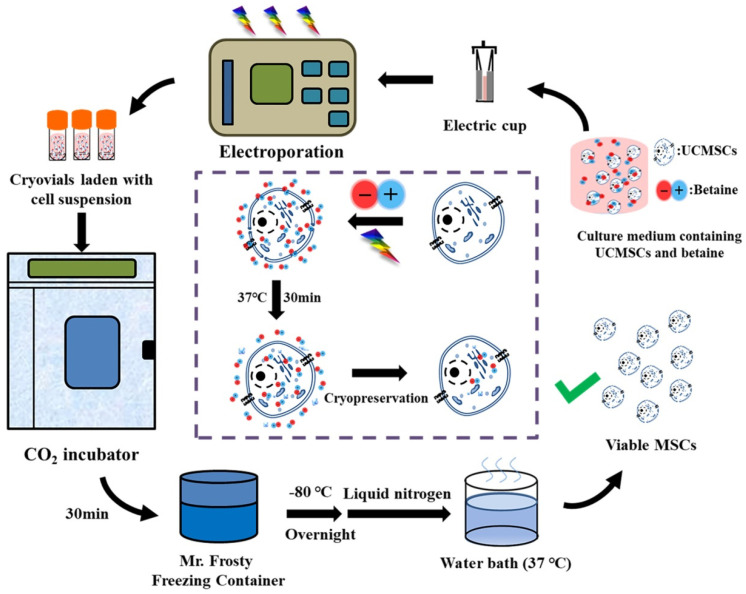
Procedure and proposed mechanism of UCMSCs cryopreservation by using betaine combined with electroporation.

The steps of cryopreservation protocol we developed based on betaine and electroporation (B–E cryopreservation) is shown in Figure 3.

### 2.3. Evaluation of UCMSCs Functions

**Figure 4:**
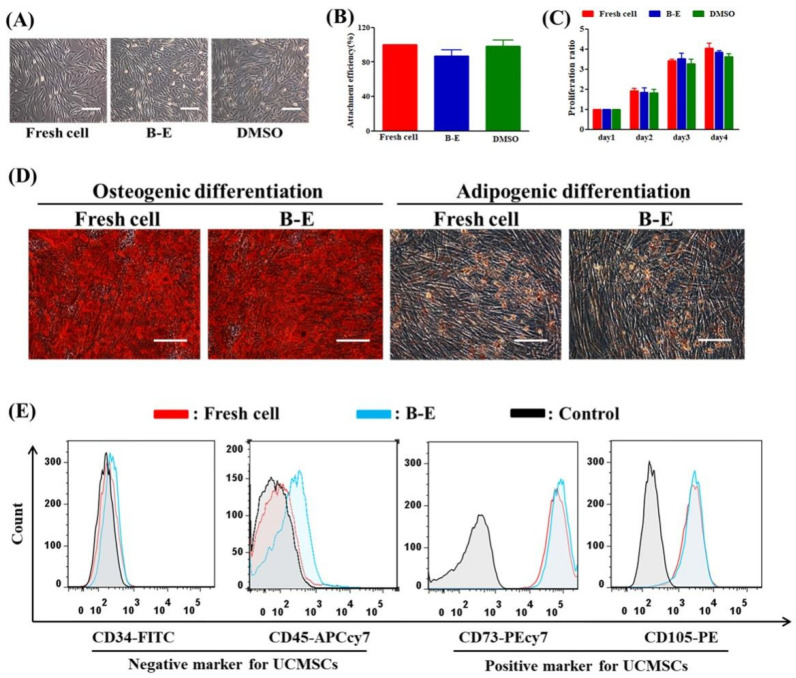
Evaluation of UCMSCs functions. (**A**) Morphology. (**B**) Attachment efficiency. (**C**) Proliferation ability. (**D**) Qualitative assessment of osteogenic and adipogenic differentiation (stained by Alizarin Red and Oil Red O, respectively). (**E**) Flow cytometry quantification of the expression of CD34 and CD45 (negative markers), CD73, and CD105 (positive markers). Control group refers to UCMSCs without cryopreservation and staining process. Scale bar = 200 μm. Value = mean ± standard deviation, n = 3.

We comprehensively characterized the in vitro biological functions of post-thaw UCMSCs. After cryopreservation, B–E cryopreservation maintained normal cell morphology and proliferation ability compared to fresh UCMSCs (Figure 4A,C). Figure 4B shows that the attachment efficiency of the UCMSCs in B–E was slightly lower than that of fresh UCMSCs or UCMSCs cryopreserved by DMSO. Further studies were conducted to assess the multipotential differentiation and the expression of specific markers of UCMSCs. After the induction culture and staining treatment, the post-thaw UCMSCs in B–E maintained similar adipogenic and osteogenic potential to fresh UCMSCs (Figure 4D). No significant difference was observed between the recovered and fresh UCMSCs in terms of expression of positive markers (CD73 and CD105) or negative markers (CD34 and CD45) (Figure 4E).

### 2.4. ROS Level of UCMSCs

**Figure 5:**
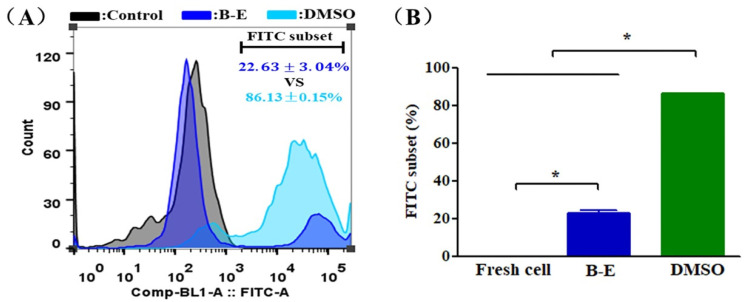
ROS level of UCMSCs. (**A**) Representative flow cytometry histograms of ROS level. (**B**) Flow cytometry quantification of FITC-positive cell population. Control group refers to UCMSCs without cryopreservation and staining process. Value = mean ± standard deviation, n = 3. * *p* < 0.05.

In this work, we assessed the antioxidant activity of betaine and DMSO to suppress the cellular overproduction of ROS caused by cryopreservation. As shown in Figure 5A,B, compared to fresh UCMSCs, an apparent increase in the ROS level was observed in post-thaw UCMSCs. However, the ROS level of UCMSCs in B–E (22.36 ± 3.04%) was significantly less than that of UCMSCs in DMSO (86.13 ± 0.15%).

### 2.5. Construction and Cryopreservation of GFP-Fluc-UCMSCs

**Figure 6:**
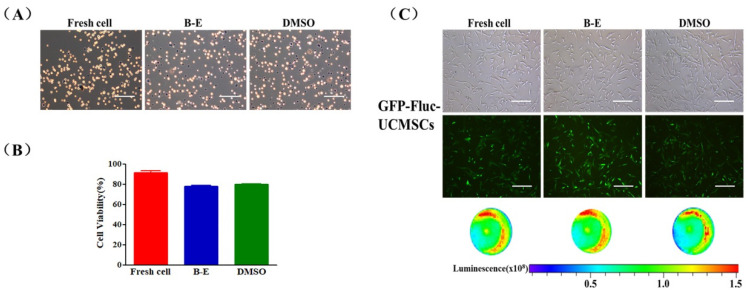
GFP-Fluc-UCMSCs cryopreservation by using betaine combined with electroporation. (**A**) Microscopic images of trypan blue stain (blue refers to dead cell, non-stain refers to live cell) and (**B**) quantification of survival rate of fresh and post thaw GFP-Fluc-UCMSCs. (**C**) Microscopic, fluorescence, and bioluminescence images of fresh and post-thaw GFP-Fluc-UCMSCs to characterize their morphology and expression of reporter gene (GFP and Fluc). Scale bar = 200μm. Value = mean ± standard deviation, n = 3.

In order to test the in vivo characteristics of UCMSCs, we constructed stable UCMSCs expressing both green fluorescent protein (GFP) and firefly luciferase (Fluc) that were called GFP-Fluc-UCMSCs by lentiviral transfection and flow cytometry sorting (Figure S1A–C). GFP-Fluc-UCMSCs showed normal morphology and adhesion ability (Figure S1D). Moreover, the transfection process did not influence the expression of surface markers and the multi-lineage differentiation potential of the GFP-Fluc-UCMSCs (Figure S1E,F). Betaine combined with electroporation was able to effectively cryopreserve GFP-Fluc-UCMSCs. The survival rate of thawed GFP-Fluc-UCMSCs reached 77.8 ± 1.55%, which was similar to the rate for DMSO cryopreservation (Figure 6A,B). The recovered GFP-Fluc-UCMSCs were able to attach to the substrate and express the GFP and Fluc genes normally (Figure 6C).

### 2.6. The In Vivo Distribution of GFP-Fluc-UCMSCs

**Figure 7:**
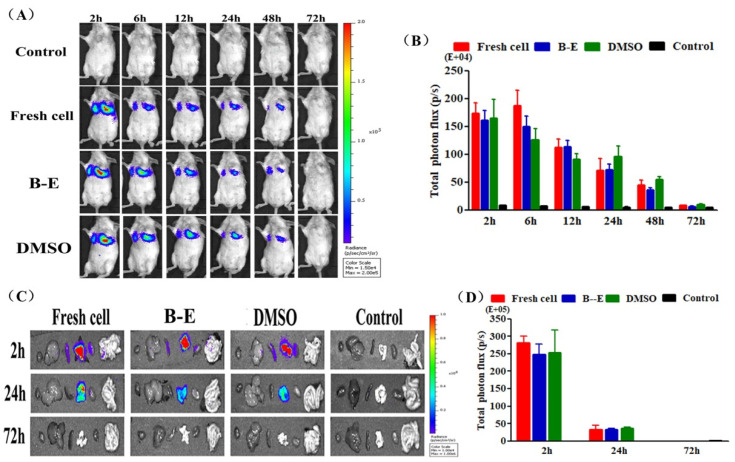
Influence of cryopreservation on in vivo distribution of GFP-Fluc-UCMSCs. (**A**) Bioluminescence images to monitor the in vivo distribution of GFP-Fluc-UCMSCs in mouse model. (**B**) Quantification of total photon flux from GFP-Fluc-UCMSCs in mouse model. (**C**) Bioluminescence images to monitor the distribution of GFP-Fluc-UCMSCs in mouse organs. The organs were arranged as heart, liver, spleen, lung, kidney, and intestines. (**D**) Quantification of total photon flux from GFP-Fluc-UCMSCs in mouse organs. Control group refers to UCMSCs without cryopreservation and transgene treatment. Fresh cell refers to GFP-Fluc-UCMSCs without cryopreservation process. DMSO refers to GFP-Fluc-UCMSCs cryopreserved by traditional protocol based on DMSO. B–E refers to GFP-Fluc-UCMSCs cryopreservation using betaine combined with electroporation at optimum condition. Value = mean ± standard deviation, n = 4.

In order to explore the influence of cryopreservation on in vivo distribution of MSCs, 5 × 10^5^ fresh or thawed GFP-Fluc-UCMSCs were intravenously administrated in mice and visualized with a bioluminescence imaging (BLI) system. As shown in Figure 7A,B, compared to fresh GFP-Fluc-UCMSCs, the in vivo distribution of the recovered GFP-Fluc-UCMSCs remained unchanged. Specifically, strong BLI signals were detected at 2 h and were concentrated in the lungs after the administration of the GFP-Fluc-UCMSCs. The signal then started to decline and returned to the baseline level at 72 h. Considering that BLI signals emitted from deeper tissues can be weakened [40], we dissected the mice and further analyzed the tissue bio-distribution of the GFP-Fluc-UCMSCs. As shown in Figure 7C,D, other than the concentration of BLI signals in the lungs, there were a small number of BLI signals distributed in the kidneys and spleen at 2 h. Then, the BLI signals began to weaken and could only be detected in the lungs. The BLI signals returned to the baseline level at 72 h.

### 2.7. Chemokine Receptor Expression and Cytoskeleton Integrity of UCMSCs

**Figure 8:**
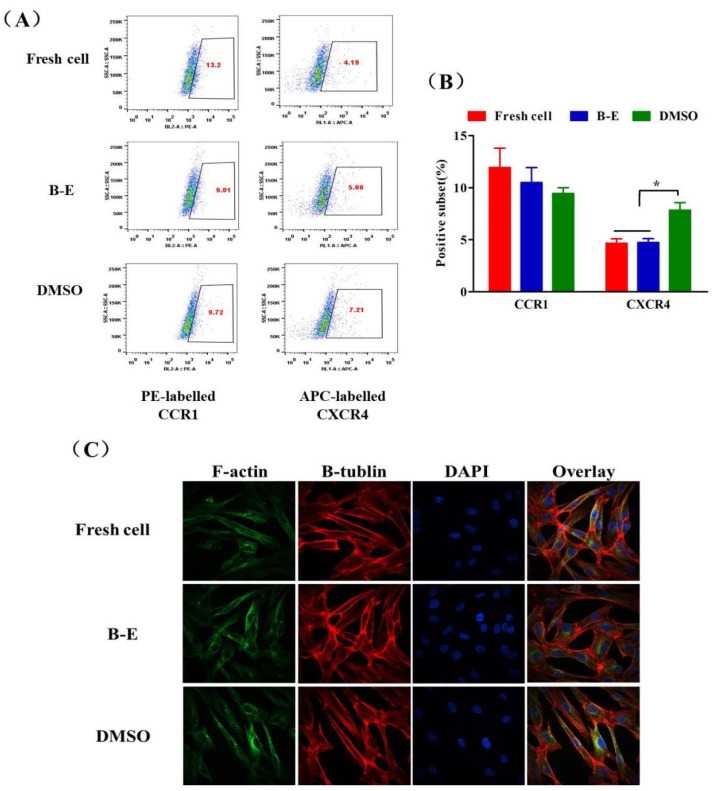
Chemokine receptor expression and cytoskeleton integrity of UCMSCs. (**A**) Representative flow cytometry dot plots and (**B**) quantification analysis of expression of CCR1 and CXCR4 of UCMSCs. Value **=** mean ± standard deviation, n = 3. (**C**) Immunostaining of F-action and β-tubulin to characterize the cytoskeleton integrity of UCMSCs. * *p* < 0.05.

In a further study, we tested the expression of CC chemokine receptor 1 (CCR1) and CXC chemokine receptor 4 (CXCR4) of cryopreserved UCMSCs that were closely related to migration and distribution [41,42,43]. As shown in Figure 8A,B, compared to fresh UCMSCs, the recovered UCMSCs from B–E cryopreservation showed normal expression of these receptors. However, DMSO cryopreservation triggered obvious upregulation of CXCR4. Though the overexpression of CXCR4 has been proven to enhance migration of MSCs [44], this effect was not reflected in the macro monitoring results (Figure 7). We also tested the cytoskeleton integrity of post-thaw UCMSCs through immune fluorescence staining. The F-actin and β-tubulin in the post-thaw UCMSCs was well-organized. No obvious differences were observed in the cytoskeleton of the recovered and fresh UCMSCs (Figure 8C).

## 3. Discussion

In this work, we aimed to achieve DMSO-free cryopreservation of UCMSCs by using natural zwitterionic molecule betaine as an alternative CPA of DMSO. It is well known that mechanical and osmotic injuries caused by ice formation during the freezing process are a major reason for cell death [45,46]. In fact, ice formation is the result of the construction of HBN among water molecules [47]. The special molecular structure of betaine, consisting of a positively charged tri-methylammonium group and a negatively charged carboxyl group, equips it with strong an electrostatic induction effect and ionic solvation effect [48]. Thus, betaine is able to strongly bind to water molecules and destroy the HBN formation [39]. Our results demonstrated that betaine decreased the freezing point and inhibited the ice formation (Figure 1A–C), which were consistent with the previous reports [28,39]. In addition, many cell types could accumulate or release intracellular betaine as an essential osmoprotectant to resist the changes in external osmotic stress [49]. The osmotic protection role of betaine was also available for UCMSCs (Figure 1D). Betaine demonstrated great potentials to protect UCMSCs from mechanical and osmotic injuries. The compatibility of CPA was critically important for cryopreservation of clinically used cell types such as MSC, red blood cells, and lymphocytes [50]. Betaine showed superior biocompatibility to DMSO in our work and was more available for UCMSC cryopreservation.

In subsequent cryopreservation experiments, incubating betaine-based cryo-medium with UCMSCs before cryopreservation for 4 h increased the viability of recovered cells to 48.07 ± 4.17% (Figure 2A), which suggested a considerable cryoprotection of betaine. However, continually increasing the betaine concentration in the cryo-medium did not improve the post thaw cell survival rate. There may exist a maximum of intracellular betaine absorbed by UCMSCs, which is likely related to the transmembrane transport manner of betaine and the expression of transporter on UCMSCs [32]. According to the result, this maximum of intracellular betaine could only provide limited protection during UCMSCs cryopreservation. To further improve efficiency of betaine for UCMSCs cryopreservation, electroporation, a commonly used molecular delivery technology, was introduced to address this problem. Under the electric stimulus within a reasonable range, the reversible hydrophobic pores form on cell membranes, enhancing permeability non-specifically [33]. Betaine molecules could quickly diffuse into cells and increase the intracellular accumulation of betaine via these temporary pores. Based on the results of many experiments, the optimal condition for UCMSCs cryopreservation (CPA concentration: 8% betaine; electroporation condition: 2.5 kV × cm^−1^, 100 μs, 8 pulses, and 1 min interval) was established. After electroporation and brief incubation, betaine could provide sufficient extracellular and intracellular protection achieving the post thaw UCMSCs survival rate of 83.45 ± 2.12% (Figure 2C), which was comparable to the efficiency of DMSO. In addition to the viability, the recovered UCSMCs demonstrated normal morphology, proliferation, differentiation potential, and expression of characteristic markers.

In normal conditions, ROS produced from energy metabolism could be balanced by a cellular antioxidant such as glutathione or antioxidases [48]. However, the drastic change in temperature during the freezing and thawing process could impair the structure integrity of mitochondria, causing the generation of excessive ROS [51,52]. The harmful ROS may disrupt the membrane structure and cause fragmentation of the chromosomes and DNA, eventually leading to cell death [53]. The ROS level in UCMSCs showed an apparent increase after cryopreservation. B–E cryopreservation significantly reduced the ROS level of recovered UCMSCs compared to DMSO cryopreservation. The antioxidative activity of betaine may explain this phenomenon. It has been reported that betaine could regulate the metabolism of sulfur amino acids such as methionine, S-adenosyl-L-methionine, and glutathione against oxidative stress [48,54]. Besides, betaine plays a significant role as chemical chaperone to stabilize the structure of macromolecules such as proteins and DNA [31,55], which is helpful to maintain the anti-oxidative activity of glutathione and antioxidases.

Apart from these primary properties tested in vitro, we should also pay attention to the in vivo distribution of UCMSCs, which is crucial in choosing the cell transplantation sites and improving the efficiency of cell transplantation [56,57]. Few studies have tested the effect of the newly established protocol on the in vivo characteristics of MSCs [11,12,15,18]. Chinnadurai et al. reported that cryopreservation impaired the cytoskeleton and altered the in vivo distribution of human MSCs. We tested the influence of B–E cryopreservation protocol on the migration and distribution of UCMSCs in mice. Fresh cell or GFP-Fluc-UCMSCs recovered from B–E cryopreservation and DMSO cryopreservation demonstrated the same in vivo distribution manner as the GFP-Fluc-UCMSCs, which were mainly concentrated in the lungs after intravenous administration and gradually diminished over 72 h. This was because lungs have a large number of microvascular circulatory systems and a relatively low blood flow rate, making it easy to capture passing cells [58]. Cytoskeleton integrity and mutual recognition of chemokine and receptor were closely related with the movement and migration of cells [24,59]. In this work, no obvious differences were observed between the two aspects of fresh UCMSCs and UCMSCs recovered from B–E cryopreservation. DMSO cryopreservation triggered obvious upregulation of CXCR4. Though the overexpression of CXCR4 has been proven to enhance migration of MSCs, this effect was not reflected in the macro monitoring results [44]. In short, betaine combined with electroporation did not alter the in vivo distribution pattern of UCMSCs.

Importantly, the novel cryopreservation protocol we developed based on betaine and electroporation exhibited a universal applicability. It maintained post thaw cell viability in different types of MSCs such as human-derived UCMSCs (83.45 ± 2.12%), mice-derived BMMSCs (84.37 ± 2.41%, in other unpublished work), and transgenic UCMSCs (77.8 ± 1.55%) recovered from B–E cryopreservation.

## 4. Materials and Method

### 4.1. Animals

Male C57bL/6 mice (6–8 weeks) were purchased from Charles River Co. (Beijing, China) and housed under Specific Pathogen Free conditions. All experimental processes were approved by the institutional ethics committee of the National Beijing Center for Drug Safety Evaluation and Research (NBCDSER, Permit No. 11-1166-3).

### 4.2. Cell Preparation

Human UCMSCs were from Linsheng Zhan’s laboratory and cultured with completed culture medium (HUXUC-90011, Cyagen Biosciences, Guangzhou, China) in a CO_2_ incubator (BB-15, Thermo Scientific, Waltham, MA, USA). Construction of GFP-Fluc-UCMSCs: UCMSCs were seeded on 24-well tissue culture polystyrene (TCPS) plates (Corning, NY, USA) overnight. Then, the medium was replaced with 2 mL completed culture medium containing 6 ug × mL^−1^ polybrene (H8761, Solarbio Science and Technology, Beijing, China). Prepared virus (pHBLV-CMV-MCS-EF1-ZsGreen-T2A-Fluc, Han biotechnology Co., Beijing, China) was added to the well, making the multiplicity of infection (MOI) values reach to 0, 1, 5, 10, 20, and 40. 2 mL fresh culture medium was supplemented in each well, and the culture medium was changed after 24 h of further culture. After transfection for 72 h, fluorescence microscope (DMI4000 B, Leica, Weztlar, Germany) was used to detect GFP gene expression. Then, 100 μL D-Fluciferin (40901ES01, Promega, Madison, WI, USA) was added to each well and incubated in the dark for 3 min, imaged with VIS Spectrum system (Lumina II, PerkinElmer, Waltham, MA, USA) to detect the Fluc gene expression. According to the result, the MOI of 40 was selected to transfect UCMSCs. The transfected UCMSCs were detached and sorted by flow cytometry (Figure S1). UCMSCs and GFP-Fluc-UCMSCs were cultured in a complete culture medium. The medium was changed every 3–4 d until the cells reached ~70–90% confluence. The adherent cells were washed with phosphate-buffered saline (PBS) (C10010500BT, Gibco, Grand Island, NY, USA), trypsinized with trypsin-EDTA (25200-056, Gibco, Grand Island, NY, USA), and centrifuged for 5 min at 500× *g*. The cell pellets were re-suspended for passaging or experimental use. Cell generation number 3–5 could be used for experimentation.

### 4.3. DSC Test

The DSC test of ice formation was performed with ultrapure water, betaine solution (*w*/*v*, 2–10%) (B2629, Sigma, St. Louis, MO, USA), and DMSO solution (*v*/*v*, 10%) (D2650, Sigma, St. Louis, MO, USA) using a DSC system (Q2000, TA, New Castle County, DE, USA). A 5–10 mg sample was weighed for the experiment. The temperature was changed from −40 °C to 10 °C at 2 °C min^−1^. The heat flow (W × g^−1^) of the sample was measured, accompanied by the appearance of an endothermic peak. The freezing point (T_f_) was the onset temperature of the heat flow curves of the samples. The relevant data are calculated as follows:

(1) $$W_{f}=\frac{\Delta \mathrm{Hsample}}{\Delta \mathrm{Hwater}}$$

(2) $$W_{\mathrm{uf}}=W_{t}-W_{f}$$

(3) $$W_{t}=W\cdot \;\frac{1}{1+m\cdot \mathrm{Ms}}$$

The integral of the curve from T_f_ to the end of the exothermic peak was the melting enthalpy (ΔH, J × g^−1^). ΔH_water_ was equal to 334 J × g^−1^. W_t_, W_f_, and W_uf_, respectively, representing the total water content, the freezing water content, and the un-freezing water content of the solution. W and m respectively refer to the mass and the molar concentration of the sample. Ms refers to the molar mass of the solute.

### 4.4. Osmotic Regulation Test

UCMSCs were seeded on 12-well TCPS plates and exposed to a fresh culture medium, culture medium + 1.25% NaCl (*w*/*v*), culture medium + 1.5% NaCl (*w*/*v*), culture medium + 1.25% NaCl (*w*/*v*) + 0.75% betaine (*w*/*v*), and culture medium + 1.5% NaCl (*w*/*v*) + 0.75% betaine (*w*/*v*) for 24 h (37 °C, 5% CO_2_). The cells were imaged by fluorescence microscope to observe the attachment and morphology of the UCMSCs.

### 4.5. Cytotoxicity

UCMSCs were seeded on 96-well TCPS plates and cultured in fresh culture medium, 2% betaine (*w*/*v*), or 2% DMSO (*v*/*v*) solution in a CO_2_ incubator for 12, 24, or 48 h. To measure cell viability, the sample solution was removed and replaced with 100 μL of fresh culture medium containing 10 μL of cell counting kit-8 (CCK-8) (CA1210, Solarbio Science and Technology, Beijing, China). Then, the samples were detected at the absorbance of 450 nm after incubation for 4 h at 37 °C. The cell viability was calculated as the percentage of cells in the sample group out of that in the control group. Meanwhile, the morphology of UCMSCs at different time points was recovered by fluorescence microscope.

### 4.6. Influence of Electroporation on Cell Viability

5 × 10^5^ UCMSCs were mixed into 300 μL of electroporation buffer, including culture medium, culture medium + 8% betaine (*w*/*v*), and culture medium + 8% DMSO (*v*/*v*). The prepared cell suspension was transferred to 2 mm electroporation cuvettes. Eight pulses of 100 μs at 1 Hz were delivered at 0, 1.5, 1.75, 2.0, 2.25, and 2.5 kV × cm^−1^. After electroporation (Multiporator, Eppendorf, Hamburg, Germany), the cell suspensions were transferred to microcentrifuge tubes (Corning, NY, USA) and incubated at 37 °C for 15 min, which allowed the cell membranes to reseal (Figure 1). The cells were collected by centrifugation and washed by PBS once. The cell survival rate was tested by the Live/Dead Kit.

### 4.7. Cell Cryopreservation

Betaine cryo-medium: a culture medium containing 0–10% betaine (*w*/*v*). DMSO cryo-medium: 90% fetal bovine serum (FBS) (*v*/*v*) mixed with 10% DMSO (*v*/*v*). All cryo-mediums were sterilized by syringe filter units (SLGP033RB-0.22 μm, Millipore, MA, USA). Routine cryopreservation: 5 × 10^5^ UCMSCs were suspended in 1 mL of cryo-medium. The cell-CPA suspension was loaded into cryovials (Corning, NY, USA), which were transferred to the Nalgene^®^ Mr. Frosty freezing container (Thermo Fisher, Waltham, MA, USA), cooling at a rate of 1 °C/min to −80 °C, stored for at least 6 h, and then transferred into liquid nitrogen. Incubation-cryopreservation: the UCMSCs suspension was incubated in a CO_2_ incubator for 4 h before routine cryopreservation. Electroporation-cryopreservation: 300 μL betaine cryo-medium laden with 5 × 10^5^ UCMSCs or GFP-Fluc-UCMSCs were electroporated at 2.25 kV × cm^−1^, eight pulses, 100 μs, and 1 Hz. The cell suspension was transferred to cryovials and replenished to 1 mL with the corresponding cryo-medium and then incubated in a CO_2_ incubator for 30 min before routine cryopreservation (Figure 1). For recovery, the cryovials were thawed in a 37 °C water bath after the indicated period.

### 4.8. Cell Viability Assay

Live/Dead Kit (L34964, Thermo Fisher, Waltham, MA, USA): the UCMSCs were re-suspended using a calcein-AM/ethidium homodimer-1 reagent mixture solution and incubated at room temperature for 30 min in darkness. A 20 μL cell suspension was then transferred to a cell counting chamber (Countstar, Shanghai, China) to photograph the live/dead staining using an inverted fluorescence microscope. Finally, the numbers of live cells (green) and dead cells (red) were measured using ImageJ. As for GFP-Fluc-UCMSCs cryopreservation, trypan blue stain (C0040, Solarbio Science and Technology, Beijing, China) was used to test cell viability. In detail, 0.4% trypan blue was mixed with cell suspension (9:1, *v*:*v*) for 3 min, then 20 μL mixture was loaded into a cell counting chamber to photograph the stain picture. Dead cells were stained with blue. The cell survival efficiency was calculated as the percentage of live cells out of the total number of cells.

### 4.9. Functional Characterization

Morphology: UCMSCs or GFP-Fluc-UCMSCs were seeded on 6-well TCPS plates and imaged after 72 h for observation of cell morphology using an inverted fluorescence microscope.

Attachment efficiency: UCMSCs were seeded overnight on 24-well TCPS plates with 300 μL of culture medium. On the following day, a 30 μL CCK-8 solution was added to each well. After incubation for 4 h at 37 °C, the absorbance at 450 nm was measured to quantify the number of cells in each sample. The attachment efficiency was calculated as the percentage of the number of cells in the post-thaw group out of the number of cells in the fresh group.

Proliferation ability: UCMSCs were seeded on 96-well TCPS plates with 100 μL of culture medium and cultured for 4 d, changing the medium every day. 10 μL of CCK-8 was added to each well, and the absorbance at 450 nm was measured to quantify the number of cells after incubation for 4 h at 37 °C. The proliferation was calculated as the ratio of the number of cells on days 2, 3, and 4 to the number of cells on day 1.

Expression of MSC markers: UCMSCs were seeded on a 60-mm TCPS plate for 72 h. The cells were detached and washed with PBS three times and were then separately stained with fluorescein (FITC) labeled CD34 (341071), Allophycocyanin-Cy7 (APCcy7) labeled CD45 (557833), phycoerythrin-Cy7 (PEcy7) labeled CD73 (5612580), and phycoerythrin (PE) labeled CD105 antibodies (562380) according to the manufacturer’s instructions. All antibodies were purchased from BioLegend (San Diego, CA, USA). APCcy7 labeled CD45 and PE labeled CD105 antibodies were used to stain GFP-Fluc-UCMSCs. These samples were tested by flow cytometer (LSR-II BD, San Diego, CA, USA) and analyzed by the FlowJo software.

Multipotential differentiation: UCMSCs or GFP-Fluc-UCMSCs were seeded on a 60-mm TCPS plate for 72 h. The cells were detached and washed with PBS three times and then reseeded on 6-well TCPS plates. The osteogenic and adipogenic induction differentiation medium kit (HUXUC-90021 and HUXUC-90031 Cyagen Biosciences, Guangzhou, China) was used to test the differential potential of UCMSCs. Oil Red O stain was used to confirm adipogenic differentiation of the UCMSCs. Alizarin Red S stain was used to confirm osteogenic differentiation. The stained cells were imaged using an inverted fluorescence microscope.

### 4.10. ROS Detection

2,7-dichlorofluorescin diacetate (DCFH-DA) (CA1410, Solarbio Science and Technology, Beijing, China) was used to detect the ROS level in post-thaw UCMSCs. Briefly, UCMSCs were washed at least once with PBS and stained with PBS containing 10 μM DCFH-DA for 30 min at 37 °C in darkness. The cell samples were tested by flow cytometer and analyzed by the FlowJo software.

### 4.11. BLI of GFP-Fluc-UCMSC Distribution In Vivo

The IVIS Spectrum system was used to image and analyze the in vivo distribution of the GFP-Fluc-UCMSCs. GFP-Fluc-UCMSCs were suspended with 500 μL of normal saline. The cell suspension was administrated intravenously to mice, which were imaged at 2, 6, 12, 24, 48, and 72 h after the intraperitoneal injection of D-Fluciferin (150 mg × kg^−1^) for 5 min. For the GFP-Fluc-UCMSC tissue distribution analysis, the mice were dissected at 2, 24, and 72 h after intraperitoneal injection of D-Fluciferin for 5 min. For imaging, the dissected tissues were arranged on black paper in the order of heart, liver, spleen, lung, kidney, and intestine.

### 4.12. Immunostaining of Cell Cytoskeleton

UCMSCs were seeded on glass-bottomed cell culture dishes (NEST, Jiangsu, China) for immunofluorescence staining of the cytoskeleton. The adherent cells were fixed with 0.4% paraformaldehyde for 30 min at room temperature and permeabilized with 0.1% Triton X-100 (T8200, Solarbio Science and Technology, Beijing, China) in PBS for 15 min at room temperature. After removing the Triton X-100, the permeabilized UCMSCs were washed three times with PBS and blocked with 5% bovine serum albumin (A8010, Solarbio Science and Technology, Beijing, China) for 1 h at room temperature. A total of 200 μL of anti-β-tublin (ab18207, Abcam, Cambridge, UK) was added, and then the UCMSCs were refrigerated overnight and washed with PBS three times. Rhodamine-conjugated phalloidin (350–555, Thermo Fisher, MA, USA) and 488-conjugated goat anti-rabbit IgG (ab150077, Abcam, Cambridge, UK) were added to the glass bottom for 60 min. The cells were washed again and incubated with a 4′-6-diamidino-2-phenylindole (DAPI) medium (C0065, Solarbio Science and Technology, Beijing, China) to stain the nuclei. The samples were imaged using confocal laser-scanning microscopy (PerkinElmer, MA, USA) and analyzed by the Volocity Demo software.

### 4.13. Chemokine Receptor Expression

UCMSCs were seeded on a 60-mm TCPS plate for 72 h. The cells were detached and washed with PBS for 3 times. UCMSCs were mixed with 100 μL PBS containing 1 mg/mL PE labeled CCR1 and APC labeled CXCR4 (5F10B29 and 12G5 BioLegend, San Diego, CA, USA), which was incubated at room temperature in dark for 15 min and then wash with PBS for 3 times. The cell samples were tested by flow cytometer and analyzed by the FlowJo software.

### 4.14. Data Analysis

Statistics and calculations were performed using were performed using the statistical software GraphPad Prism 6 (San Diego, CA, USA). The data are presented as the mean ± SD. Significance (* *p* < 0.05) was determined with one-way ANOVA or a two-tailed homoscedastic Student’s t-test at a significance level of 0.05. The results were from at least three independent runs.

## 5. Conclusions

In conclusion, we established a DMSO-free cryopreservation protocol for UCMSC based on betaine and electroporation. This cryopreservation method exhibited superior biocompatibility and cryopreservation efficiency. Betaine as a novel CPA could protect UCMSCs from cryo-injuries including ice and osmotic injuries. Moreover, because of the strong anti-oxidative activity of betaine, UCMSCs recovered from B–E cryopreservation showed less ROS production and reduced oxidative stress upon thawing. Finally, B–E cryopreservation made the post thaw cell viability reach to 84.37 ± 2.41%, which was comparable to the efficiency of DMSO cryopreservation. These recovered UCMSCs maintained normal in vitro functions like proliferation, multi-line differentiation, and expression of MSC markers. Besides, B–E cryopreservation did not influence the in vivo distribution of UCMSCs. This work provides a DMSO-free cryopreservation technology for highly efficient cryopreservation of MSCs.

## Data Availability

All data generated in this study are included in this published article and its Supplementary information files.

## Supplementary Materials

The following are available online at https://www.mdpi.com/article/10.3390/ijms22147445/s1.
