# Correction: Gao et al. Dimethyl Sulfoxide-Free Cryopreservation of Human Umbilical Cord Mesenchymal Stem Cells Based on Zwitterionic Betaine and Electroporation. *Int. J. Mol. Sci.* 2021, *22*, 7445

**DOI:** 10.3390/ijms27198442

**Published:** 2026-09-22

**Authors:** Lei Gao, Qianqian Zhou, Yulong Zhang, Sujing Sun, Liping Lv, Ping Ma, Jing Yang, Min Liu, Lei Zhang, Xiaohui Wang, Linsheng Zhan

**Affiliations:** 1 Department of Emerging Transfusion Technology, Institute of Health Service and Transfusion Medicine, Academy of Military Medical Sciences, Beijing 100850, China; gl8023jc@163.com (L.G.); zhouqianvv521@126.com (Q.Z.); zhangyulongklg@163.com (Y.Z.); ammsreb@163.com (S.S.); lvlp2007@sina.com (L.L.); maping1111@hotmail.com (P.M.); 2 Department of Biochemical Engineering, School of Chemical Engineering and Technology, Tianjin University, Tianjin 300350, China; jing_yang@tju.edu.cn (J.Y.); minliu@tju.edu (M.L.); 3 Frontier Science Center for Synthetic Biology and Key Laboratory of Systems Bioengineering (MOE), School of Chemical Engineering and Technology, Tianjin University, Tianjin 300350, China; 4 Qingdao Institute for Marine Technology of Tianjin University, Tianjin University, Qingdao 266235, China; 5 Key Laboratory of Advanced Energy Materials Chemistry, Ministry of Education, College of Chemistry, Nakai University, Tianjin 300071, China

In the original publication [1], there was a mistake in Figure 7 as published. In Figure 7A, due to an inadvertent error, the same mouse image was used twice. The corrected Figure 7 appear below. The authors state that the scientific conclusions are unaffected. This correction was approved by the Academic Editor. The original publication has also been updated.

## Figures and Tables

**Figure 7:**
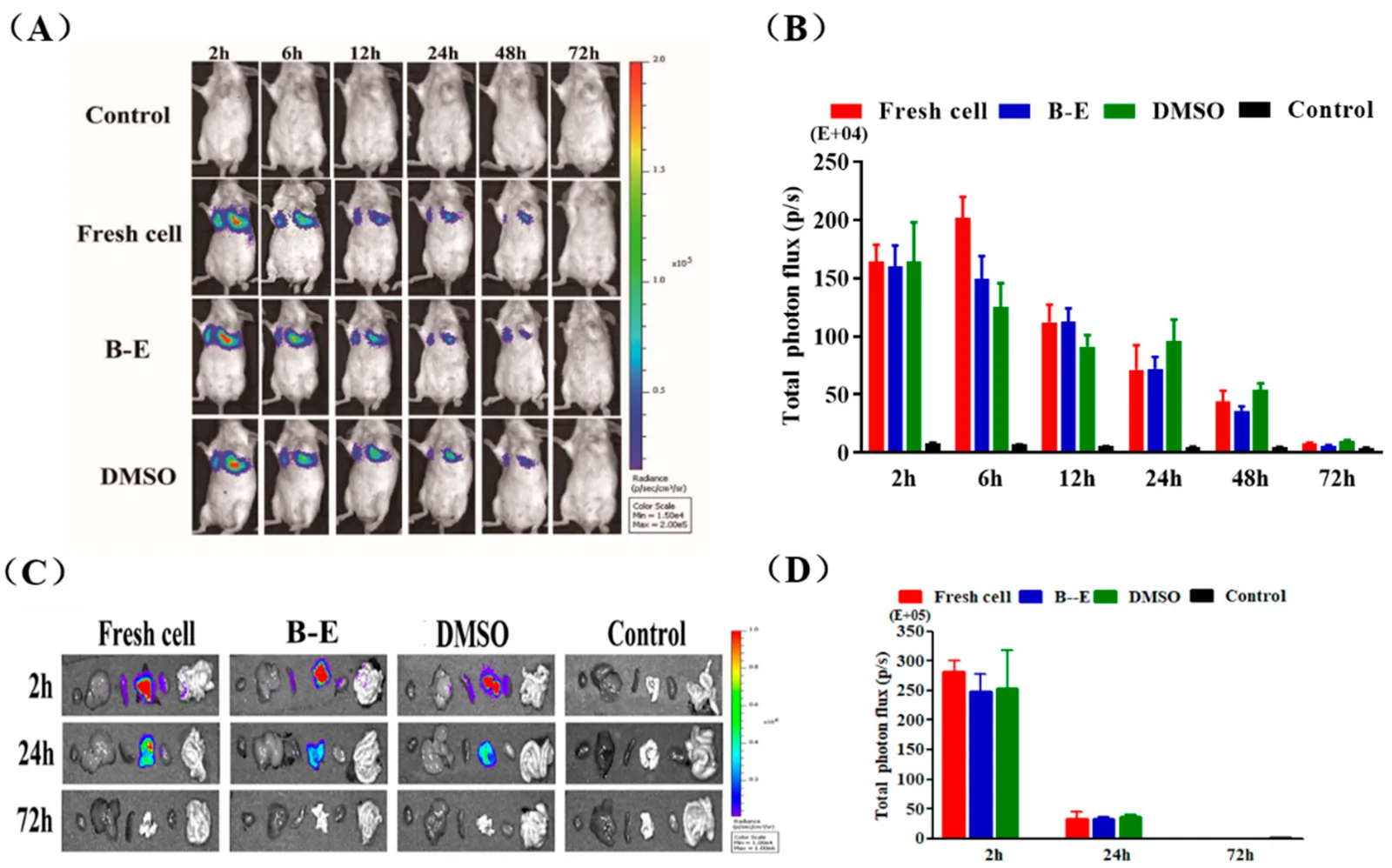
Influence of cryopreservation on in vivo distribution of GFP-Fluc-UCMSCs. (**A**) Bioluminescence images to monitor the in vivo distribution of GFP-Fluc-UCMSCs in mouse model. (**B**) Quantification of total photon flux from GFP-Fluc-UCMSCs in mouse model. (**C**) Bioluminescence images to monitor the distribution of GFP-Fluc-UCMSCs in mouse organs. The organs were arranged as heart, liver, spleen, lung, kidney, and intestines. (**D**) Quantification of total photon flux from GFP-Fluc-UCMSCs in mouse organs. Control group refers to UCMSCs without cryopreservation and transgene treatment. Fresh cell refers to GFP-Fluc-UCMSCs without cryopreservation process. DMSO refers to GFP-Fluc-UCMSCs cryopreserved by traditional protocol based on DMSO. B–E refers to GFP-Fluc-UCMSCs cryopreservation using betaine combined with electroporation at optimum condition. Value = mean ± standard deviation, n = 4.
