# Supplementary material for: Dimethyl Sulfoxide-Free Cryopreservation of Human Umbilical Cord Mesenchymal Stem Cells Based on Zwitterionic Betaine and Electroporation
Source: Int J Mol Sci. 2021 Jul 12;22(14):7445. doi: 10.3390/ijms22147445 (PMC8306716; doi:10.3390/ijms22147445)
Supplement: Supplementary file 1 [file ijms-22-07445-s001.zip › ijms-1269354-Supplementary Materials.pdf]

Supporting Information

Dimethyl Sulfoxide-Free Cryopreservation of Human Umbilical Cord Mesenchymal Stem Cells Based on Zwitterionic Betaine and Electroporation

Lei Gao[a,b,c,d], Qianqian Zhou[a], Yulong Zhang[a], Sujing Sun[a], Liping Lv[a] , Ping Ma[a], Jing Yang[b,c,d], Min Liu[b,c,d], Lei Zhang[b,c,d,*], Xiaohui Wang[a,*], Linsheng Zhan[a,*]

[a]Department of Emerging Transfusion Technology, Institute of Health Service and Transfusion Medicine, Academy of Military Medical Sciences, Beijing, 100850, People's Republic of China

[b]Department of Biochemical Engineering, School of Chemical Engineering and Technology, Tianjin University, Tianjin 300350, P.R. China

[c]School of Chemical Engineering and Technology, Frontier Science Center for Synthetic Biology and Key Laboratory of Systems Bioengineering (MOE), Tianjin University, Tianjin 300350, P. R. China

[d]Qingdao Institute for Marine Technology of Tianjin University, Qingdao 266235, P. R. China

[*]Corresponding Author:

E-mail: Lei Zhang: lei_zhang@tju.edu.cn
Xiaohui Wang: lovechina1980@163.com
Linsheng Zhan: lszhan91@yahoo.com

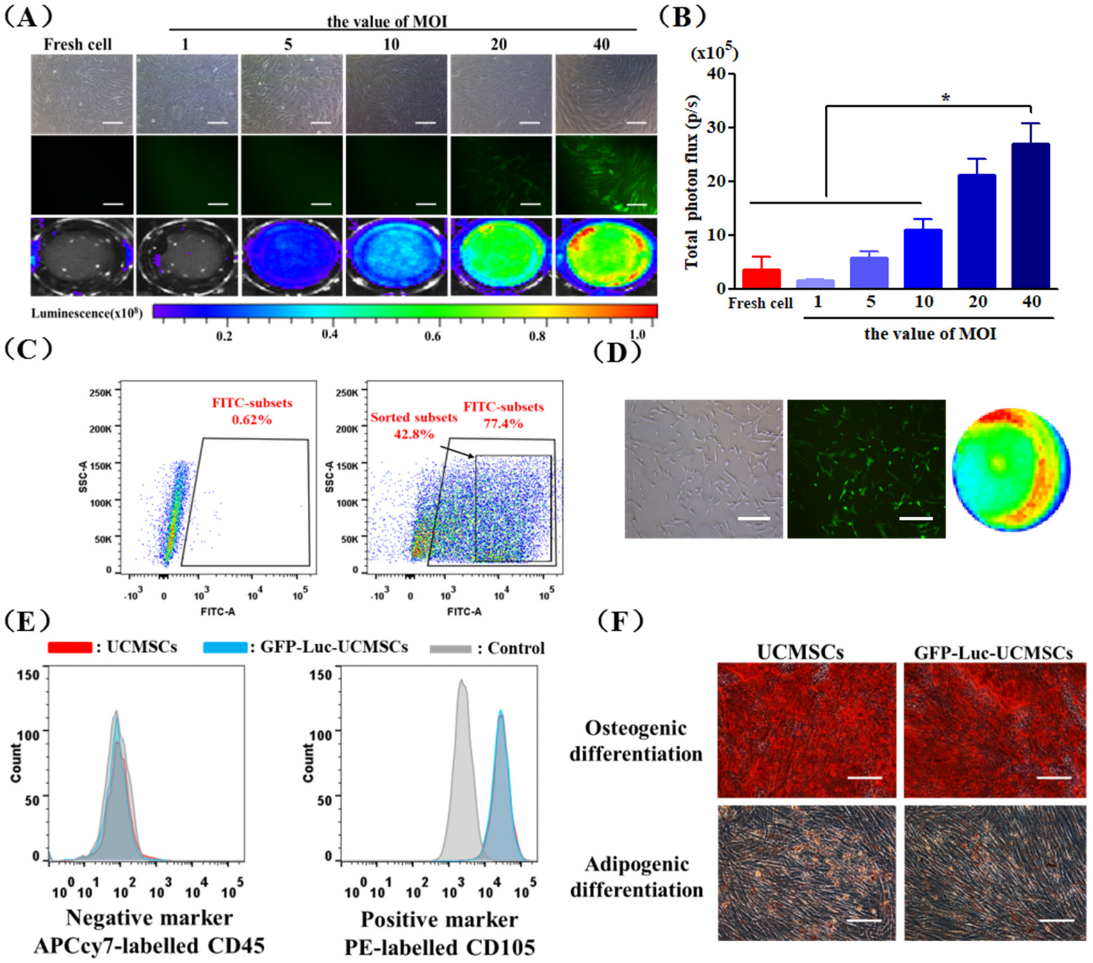

Figure S1. Construction of GFP-Fluc-UCMSCs. (A) Fluorescent images and bioluminescence images of UCMSCs transfected at different MOI value. (B) Bioluminescence intensity of UCMSCs transfected at different MOI value. (C) Flow cytometry dot plots of GFP-Fluc-UCMSCs sorting. (D) Microscopic images, fluorescent images and bioluminescence images of GFP-Fluc-UCMSCs. (E) CD45/105 expression of UCMSCs and GFP-Fluc-UCMSCs. (F) Osteogenic and adipogenic differentiation of UCMSCs and GFP-Fluc-UCMSCs. Scale bar= 200 μm. Value = mean ± standard deviation, n ⩾ 3. *$P < 0.05$.
